# Adenovirus and *Mycoplasma pneumoniae* co-infection as a risk factor for severe community-acquired pneumonia in children

**DOI:** 10.3389/fped.2024.1337786

**Published:** 2024-01-31

**Authors:** Qihong Chen, Lihua Lin, Ning Zhang, Yungang Yang

**Affiliations:** ^1^ Department of Pediatrics, The First Affiliated Hospital of Xiamen University, Xiamen, China; ^2^ Pediatric Key Laboratory of Xiamen, Xiamen, China

**Keywords:** community-acquired pneumonia, etiology, adenovirus, *Mycoplasma pneumoniae*, risk factor, children

## Abstract

**Background:**

To investigate the pathogenic characteristics and risk factors of pediatric severe community-acquired pneumonia (CAP).

**Methods:**

We retrospectively analyzed the clinical data of hospitalized children with severe CAP from April 2014 to June 2019 in China. Data of age, sex and pathogenic results were collected: bacterial and fungal cultures, respiratory viruses from sputum or bronchoalveolar lavage fluid (BALF), serum *Mycoplasma pneumoniae* (MP)-IgM and *Chlamydia Pneumoniae*-IgM, and BALF or blood (1-3)-β-D-glucan/galactomannan test.

**Results:**

A total of 679 children with severe CAP were included in the analysis. The number of cases infected with MP was higher in males than in females. There were significant differences between the ≤1-year and >1-year groups in terms of pathogen. The top three bacteria cultured were *Haemophilus influenzae* (57/679, 8.4%), *Streptococcus pneumoniae* (50/679, 7.4%), and *Pseudomonas aeruginosa* (25/679, 3.7%). The top three viruses detected were adenovirus (AdV, 124/679, 18.3%), respiratory syncytial virus (24/679, 3.5%), and parainfluenza virus (21/679, 3.1%). AdV and MP were the leading pathogens, detected in 18.3% and 32.6% cases, respectively. MP infection increased the risk of AdV infection (OR 3.77, *p* < 0.0001). MP infection was a risk factor for severe AdV-infected pneumonia, while sex, age, bacteria, *Chlamydia Pneumoniae*, fungal, and AdV infections were risk factors for severe MP-infected pneumonia.

**Conclusions:**

AdV and MP were dominant pathogens in children with severe CAP. AdV and MP infection predisposed each other to develop severe illness. AdV-MP co-infection may lead to severe pneumonia.

## 1 Introduction

Community-acquired and hospital-acquired pneumonia are common in children. In particular, pneumonia often has an acute onset and progress rapidly, is amongst the major causes of under-five mortality (1). The high mortality in children is partly due to an immature immune system and poor mucociliary clearance to excrete pathogens. Aside from that, highly pathogenic microbes predispose patients to severe community-acquired pneumonia (CAP).

Main pathogens of severe CAP were estimated to be viruses (61%) and bacteria (27%), whereas *Mycoplasma pneumoniae* (MP) accounted for only 1% of causes, according to a study in children from Asia and Africa (2). However, MP pneumonia was reported in 38% of CAP children in North China, with cyclic epidemics occuring every 2–3 years (3). Moreover, reports of MP pneumonia in children under the age of 5 have been increasing year by year. In addition to respiratory symptoms such as fever and cough, it can also be accompanied by pleural effusion, atelectasis, and in severe cases, even septic shock leading to death (4). Meanwhile, in 2019, a regional outbreak of AdV was reported in South China, with positive rate peaking at 79% of severe CAP children (5). ADV infection is one of the main pathogens causing severe viral pneumonia in children, often occurring in children under 5 years old. Some children have severe clinical manifestations and are prone to multiple systemic complications. Severe cases are prone to chronic respiratory diseases, which is one of the important causes of death and disability in infants and young children with pneumonia (6). Moreover, mixed infection was common in CAP children, and was related to higher disease severity than single pathogen (7, 8). Specifically, virus-bacterial coinfections were detected in 4%–35% of CAP children (7, 8), virus-MP coinfections in 27% of cases (9).

Early prediction and diagnosis of severe cases is crucial in CAP management. Numerous studies provided clinical factors to predict disease deterioration to severe CAP, such as hypoxemia, age, vital signs, radiographic features, and comorbidity (10, 11). Due to a varied pathogenicity, etiology—such as genotypes of pathogens and coinfection of pathogens—plays a major role in disease severity (7, 8, 12). However, the effect of coinfection on disease severity is not well understood. Considering the recently increased prevalence of AdV and MP, the influence of AdV-MP coinfection on severity is of interest.

Therefore, this study retrospectively analyzed the clinical data of 679 children with severe CAP, in hopes of uncovering the effect of AdV-MP coinfection on disease severity, and aid in predicting severe CAP. Further, since etiology of CAP varied among regions, the pathogen results in our study provide information on etiology of pediatric CAP in South China.

## 2 Methods

### 2.1 Eligibility criteria

This retrospective study was carried out in the pediatric department of the First Affiliated Hospital of Xiamen University (Fujian Province, China) from April 2014 to June 2019. Children with severe CAP who were hospitalized and underwent bronchoalveolar lavage, aged 28 days to 14 years, were enrolled in this study. A total of 679 children were enrolled. Our study protocol was approved by the Human Research Ethics Committee of the First Affiliated Hospital of Xiamen University. Guardians of all participants signed the written informed consent at the time of hospital admissions.

### 2.2 Diagnostic criteria for severe CAP

In this study, the diagnostic criteria for severe CAP were in accordance with the *Guidelines for Management of Community-Acquired Pneumonia in Children (revised in 2013)* issued by the Subspecialty Group of Respiratory Diseases, Chinese Society of Pediatrics (13). Pediatric patients with CAP who fulfilled any of the following criteria were diagnosed as severe pneumonia: poor general condition, refused eating or having signs of dehydration, impaired consciousness, elevated respiratory rate (infant >70 breaths/minute, older child >50 breaths/minute), purpura, respiratory distress (groan; nasal flaring; and three retraction signs, i.e, intercostal retractions, substernal retractions, and suprasternal retractions), multilobar involvement or ≥2/3 of the lung involved, pleural effusion, ≤92% percutaneous oxygen saturation, and extra-pulmonary complications (13).

### 2.3 Collection of clinical variables

Patients’ age and sex were collected for statistical analysis.

### 2.4 Collection of specimens

#### 2.4.1 Collection of nasopharynx specimen

For all patients, the nasopharynx specimens were collected using nasopharyngeal swabs.

#### 2.4.2 Sputum collection

For patients who needed non-invasive mechanical ventilation, the nasopharyngeal secretion was collected with a disposable sterile suction tube. For patients who needed invasive mechanical ventilation, sputum was collected from an endotracheal tube using negative pressure aspiration, and placed in sterile test tubes.

#### 2.4.3 Bronchoscopy and bronchoalveolar lavage fluid (BALF) collection

Patients were fasted for six hours before the procedure, and were then given atropine (0.01 mg/kg) to reduce airway secretion, an intravenous injection of Midazolam (0.1 mg/kg) for sedation, and 2% of lidocaine for surface anesthesia on the patient's throat skin. The bronchoscope of the Olympus BF-XP60 model or the Olympus BF-MP60 model was selected according to patient age, and 3–5 ml/kg of sterile physiological saline at 37°C was then injected through the selected bronchoscope and bronchoalveolar lavage fluid (BALF) was collected. Bronchoscopy was performed in accordance with the operating specifications.

The above samples were sent for examination within 30 min. The pathogen test was performed immediately after confirming the specimens were qualified.

### 2.5 Detection of pathogens

#### 2.5.1 Respiratory virus detection of nasopharynx and BALF specimens

Nasopharynx and BALF specimens were tested for respiratory viruses. For some specimens, respiratory virus antigens (comprising influenzae viruses A and B, parainfluenza viruses 1, 2, and 3, RSV, and AdV) were detected by direct immunofluorescence assay kits (Diagnostic Hybrids, Inc., San Diego, CA). In other specimens, respiratory viruses (comprising type A influenzae virus, H_1_N_1_ type A influenzae virus, H_3_N_2_ type A influenzae virus, parainfluenza virus, metapneumovirus, RSV, AdV, rhinovirus, bocavirus, type B influenzae virus, and coronavirus) were detected by PCR-capillary electrophoresis fragment analysis (Respiratory pathogen multiplex assay kit, Health Gene Technologies Co., Ltd., Zhejiang Province, China). All kits were used according to the manufacturers’ instructions.

#### 2.5.2 Bacterial and fungal cultures of sputum and BALF

The sputum and BALF specimens were tested for bacterial and fungal culture. Specimens were prepared as suspensions, followed by the inoculation of the suspensions to the culture dishes, which were placed in an incubator containing 5%–8% carbon dioxide at 37°C. The cultures and the identification of the bacteria and fungi were performed according to the pathogen test procedures.

#### 2.5.3 *Mycoplasma pneumoniae*-specific immunoglobulin M (MP-IgM) and *Chlamydia Pneumoniae*-specific immunoglobulin M (CP-IgM) detection of serum

For all patients, venous blood samples were detected for MP-IgM and CP-IgM. MP-IgM was detected via the Diagnostic Kit for Measurement of Antibodies to *Mycoplasma pneumoniae* (Passive Particle Agglutination; Fujirebio, Japan), CP-IgM via Anti-*Chlamydia pneumoniae* ELISA (IgM; EUROIMMUN Medizinische Labordiagnostika AG, Germany), according to the manufacturers’ instructions. A CP-IgM result of ≥1.1 ratio was considered positive. The diagnostic criteria for Mp infection are serum single MP-IgM titer ≥160 and positive MP nucleic acid test or MP-IgM titer increased or decreased by 4 times or more in the recovery and acute phases.

#### 2.5.4 Fungal detection of BALF and blood samples

The BALF and blood samples were tested for fungi by two spectrophotometric assays: the (1-3)-β-D-glucan assay (G assay, Zhanjiang A & C Biological Ltd., Guangdong Province, China; for a fungal cell wall component, a result of <100.5 pg/ml glucan was considered negative, >100.5 pg/ml glucan positive) and the Platelia™ Aspergillus Ag assay (GM assay, Bio-Rad; a value of the *Aspergillus* galactomannan antigen >0.5 *μ*g/L or >1.0 *μ*g/L was considered positive for serum or BALF, respectively). Both assays were performed according to the manufacturers’ instructions. A positive result in either the G assay or GM assay was considered as fungal infection in the samples.

### 2.6 Statistical analysis

SPSS 25.0 statistical software (IBM SPSS, Chicago, IL) was used for statistical analysis. The categorical variables were presented as the number of cases (*n*) or/and percentage (%), compared by Fisher's exact test with odds ratio (OR). Risk factors were analyzed by multivariate logistic regression analysis. A two-sided *p*-value less than 0.05 was considered statistically significant.

## 3 Results

First, to identify the relationship between age and sex, we analyzed the data of 679 pediatric patients with severe CAP. All 679 patients comprised 440 (64.80%) males and 239(35.20%) females. The patients ≤1-year-old were 266 cases, comprising 173 (65.04%) males and 93 (34.96%) females; and those >1-year-old were 413 cases, comprising 267 (64.65%) males and 146 (35.35%) females. No significant difference in sex was found between the two age groups (*χ^2^ *= 0.011, *p* = 0.918).

Second, to identify the relationship between the etiology and sex, we categorized all patients based on sex, then compared them with the presence of each pathogen infection (Table 1). The patients with positive bacterial, viral, MP, CP, and fungal infections accounted for 30.04%, 25.92%, 32.55%, 5.74%, and 7.95%, of the total cases, respectively. Male patients had a significantly lower risk of MP infection (29.3% in males vs. 38.5% in females; *p* = 0.016, OR 0.66). Meanwhile, bacterial, viral, CP, and fungal infections had no significant association with the patient's sex.

**Table 1:** The impact of sex on etiology of children with severe CAP.

Pathogen	Male	Female	Odds ratio (95% CI)	*p* value
Bacteria				
Positive	136 (66.67%)	68 (33.33%)	1.13 (0.79–1.60)	0.540
Negative	304 (64.0%)	171 (36.0%)		
Virus				
Positive	119 (67.61%)	57 (32.39%)	1.18 (0.82–1.70)	0.409
Negative	321 (63.82%)	182 (36.18%)		
MP				
Positive	129 (58.37%)	92 (41.63%)	0.66 (0.48–0.92)	0.016^*^
Negative	311 (67.90%)	147 (32.10%)		
CP				
Positive	25 (64.10%)	14 (35.90%)	0.97 (0.49–1.88)	>0.999
Negative	415 (64.84%)	225 (35.16%)		
Fungi				
Positive	33 (61.11%)	21 (38.89%)	0.84 (0.47–1.46)	0.555
Negative	407 (65.12%)	218 (34.88%)		

MP, *Mycoplasma pneumoniae*; CP, *Chlamydia Pneumoniae*.

* *p *< 0.05.

Since age was reported to affect the incidence of pediatric severe CAP (11), we categorized all patients based on age (≤ 1-year-old or >1-year-old), then compared them with the presence of each pathogen infection (Table 2). Compared with those >1-year-old patients, the ≤1-year-old patients had a significantly higher rate of CP infection (OR 2.10, *p* = 0.028) and bacterial infection (OR 1.64, *p* = 0.004), whereas a significantly lower rate of viral infection (OR 0.63, *p* = 0.012) and MP infection (OR 0.10, *p* < 0.00001).

**Table 2:** The impact of age on etiology of children with severe CAP.

Pathogen	≤1 year	>1 year	Odds ratio (95% CI)	*p* value
Bacteria				
Positive	97 (47.55%)	107 (52.45%)	1.64 (1.18–2.29)	0.004^*^
Negative	169 (35.58%)	306 (64.42%)		
Virus				
Positive	55 (31.25%)	121 (68.75%)	0.63 (0.44–0.91)	0.012^*^
Negative	211 (41.95%)	292 (58.05%)		
MP				
Positive	22 (9.95%)	199 (90.05%)	0.10 (0.06–0.15)	<0.0001^*^
Negative	244 (53.28%)	214 (46.72%)		
CP				
Positive	22 (56.41%)	17 (43.59%)	2.10 (1.08–4.05)	0.028^*^
Negative	244 (38.13%)	396 (61.87%)		
Fungi				
Positive	24 (44.44%)	30 (55.56%)	1.27 (0.72–2.24)	0.468
Negative	242 (38.72%)	383 (61.28%)		

MP, *Mycoplasma pneumoniae*; CP, *Chlamydia Pneumoniae*.

* *p *< 0.05.

**Figure 1:**
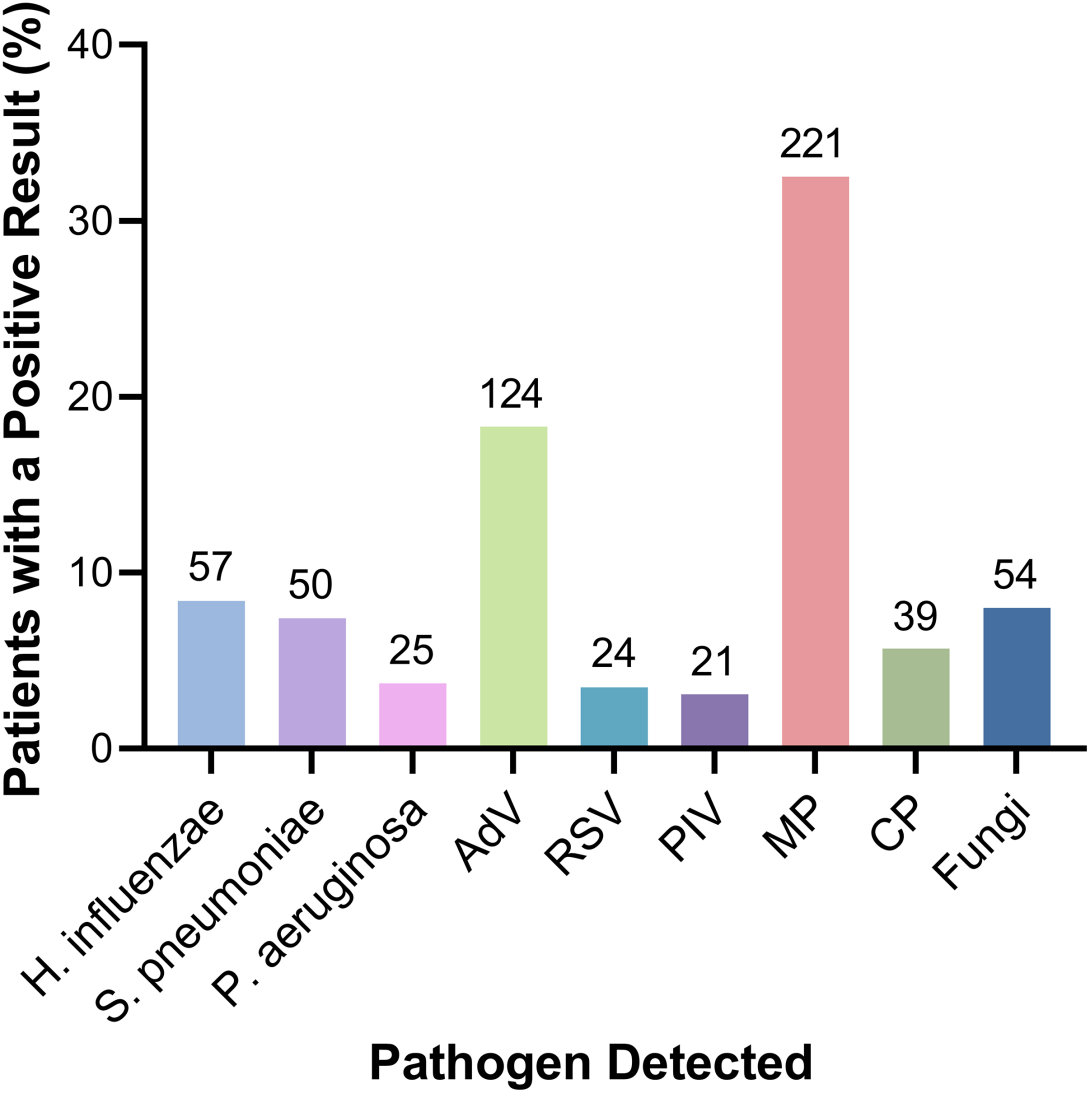
The proportion of detected specific pathogens in children with severe CAP.

Further, we focused on the most commonly detected pathogens (Figure 1). The top three bacteria in the culture were *Haemophilus influenzae* (57/679, 8.4%), *Streptococcus pneumoniae* (50/679, 7.4%), and *Pseudomonas aeruginosa* (25/679, 3.7%). The top three viruses detected were AdV (124/679, 18.3%), RSV (24/679, 3.5%), and parainfluenza virus (21/679, 3.1%). Note that the case numbers of AdV and MP infections were 124 (124/679, 18.3%) and 221 (221/679, 32.6%), respectively, which were markedly higher than that of any other pathogen infections (Figure 1).

We analyzed the relation between AdV and other pathogen infections (Table 3). Patients infected with MP demonstrated a higher risk of AdV infection than those without MP infection (OR 3.77, *p* < 0.0001), whereas other pathogens were not found to affect the incidence of AdV infection.

**Table 3:** Relation between AdV and other pathogen infections in children with severe CAP.

Pathogen	AdV positive	AdV negative	Odds ratio (95% CI)	*p* value
Bacteria				
Positive	38 (18.63%)	166 (81.37%)	1.04 (0.68–1.58)	0.914
Negative	86 (18.11%)	389 (81.89%)		
MP				
Positive	72 (32.58%)	149 (67.42%)	3.77 (2.54–5.57)	<0.0001^*^
Negative	52 (11.35%)	406 (88.65%)		
CP				
Positive	3 (7.69%)	36 (92.31%)	0.36 (0.11–1.06)	0.089
Negative	121 (18.91%)	519 (81.09%)		
Fungi				
PosItive	14 (25.93%)	40 (74.07%)	1.64 (0.88–3.07)	0.142
Negative	110 (17.60%)	515 (82.40%)		

AdV, adenovirus; MP, *Mycoplasma pneumoniae*; CP, *Chlamydia Pneumoniae*.

* *p *< 0.05.

Lastly, to evaluate the risk factors of severe AdV-infected pneumonia, multivariate logistic regression analysis was carried out by using AdV infection as an independent variable, and patient's age, sex, bacteria, MP, CP, and fungi as covariates (Table 4). The analysis showed that MP-positive infection was a risk factor for AdV infection (OR = 0.279, *p* = 0.000), while no significant correlation was found between the AdV infection and other factors, including patient's sex, age, bacteria, CP, and fungi. To further verify the correlation of AdV and MP infection, we used MP infection as an independent variable and patient's sex, age, bacteria, fungi, CP, and AdV as covariates to identify the risk factors affecting MP infection (Table 5). The results showed that AdV infection, together with the patient's sex, age, bacterial, and CP infections were risk factors for MP infection (OR = 0.28, 1.71, 10.31, 1.68, and 0.33, respectively, *p *< 0.05), whereas no significant correlation was found between MP infection and fungal infection.

**Table 4:** Multivariate analysis of factors associated with AdV infection.

	Odds ratio (95% CI)	*p* value
Sex	0.81 (0.53–1.23)	0.337
Age	1.42 (0.85–2.35)	0.179
Bacteria	0.83 (0.53–1.32)	0.435
MP	0.28 (0.18–0.44)	0.000^*^
CP	3.07 (0.90–10.43)	0.072
Fungi	0.57 (0.29–1.14)	0.111

AdV, adenovirus; CI, confidence interval; MP, *Mycoplasma pneumoniae*; CP, *Chlamydia Pneumoniae*.

* *p *< 0.05.

**Table 5:** Multivariate analysis of factors associated with MP infection.

	Odds ratio (95% CI)	*p* value
Sex	1.71 (1.17–2.52)	0.006^*^
Age	10.31 (6.25–17.03)	0.000^*^
Bacteria	1.68 (1.09–2.58)	0.018^*^
Fungi	1.21 (0.59–2.48)	0.607
CP	0.33 (0.15–0.75)	0.008^*^
AdV	0.28 (0.18–0.44)	0.000^*^

AdV, adenovirus; CI, confidence interval; MP, *Mycoplasma pneumoniae*; CP, *Chlamydia Pneumoniae*.

* *p *< 0.05.

## 4 Discussion

We analyzed the etiology of pediatric severe CAP between the year 2014 and 2019 in Xiamen, South China. We observed that AdV and MP were the predominant pathogens. MP coinfection was proved to be a risk factor of severe AdV pneumonia. Likewise, AdV coinfection was among the risk factors of severe MP pneumonia.

The positive rate of AdV (18%) or MP (33%) was notably higher than any other pathogen (Figure 1). This rate is higher than that in worldwide CAP children. A meta-analysis was conducted from 152,209 CAP children worldwide, and reported that viruses were detected in 55%, including 7% cases infected with AdV (14). Pathogens of CAP following viruses are atypical pathogens (accounting for 10% cases) and bacteria (5% cases) (7, 15–17). Moreover, pathogens are more common in severe CAP children: viruses and bacteria were detected in 68% and 23% cases, respectively (2). A concurrent local study revealed that AdV was detected in 9% of severe CAP children using the traditional direct immunofluorescence assay (18). In contrast, another concurrent study in South China reported that MP and AdV were detected in 97% and 79% of severe CAP children, respectively, using a multiplex PCR assay (4). The high positive rate of AdV and MP in this study may be due to the prevalence of pathogens among severe CAP children, as well as a high sensitivity of the multiplex PCR assay which was used upon available.

The prevalence of AdV (18%, Figure 1) in this study indicates a rising incidence of AdV infection in severe CAP children in South China. A study collected data of 161 079 Chinese children diagnosed with acute respiratory illness between the year 2010 and 2021, and revealed a baseline AdV positive rate of 6%, and peaked at 10%–14% every five years (19). In line with the increased prevalence of AdV, the severity of AdV-induced CAP surged during the year 2018 and 2019, when 34% of hospitalized AdV-infected children had severe CAP (20), with a mortality rate of 2.8% among AdV-infected acute respiratory illness children (19). The high pathogenicity could be explained by a shift of dominant subtype from AdV-3 to AdV-7 (19). Compared to AdV-3, AdV-7 showed promoted replication and induced stronger cytokine response, thus causing a more severe airway inflammation and a longer duration of viral shedding (21–23). Eventually, patients infected with AdV-7 presented with a more severe phenotype: a longer fever, an increased incidence of severe pneumonia and admission to an intensive care unit, and a longer hospital stay (12, 23–25).

Clinically, severe AdV-induced pneumonia occurs frequently in children. Not only that AdV-infected patients presented a high rate of severe illness (17%–38%) (12, 24, 25), patients with severe AdV pneumonia also had a high mortality rate of 16%–26% (26–28), and are prone to disseminated complications such as shock and acute respiratory distress syndrome (27). Survivors may still suffer from non-reversible lesions, such as bronchiolitis obliterans in 24% (34/139) severe patients (29) and bronchiectasis (30). Thus, healthcare providers need to beware of the potential severity of adenovirus pneumonia and signs of disease progression.

The observed high incidence of MP infection (33%, Figure 1) is consistent with a previous study on 27 498 patients in North China (5). *Mycoplasma pneumoniae* pneumonia (MPP) is mostly mild, even severe MPP may be self-limited with reversible pathologic changes (31). However, the incidence of severe MPP surged from 1% to 35% during the past decade in China (5). In the US, 12% of hospitalized MPP children required intensive care (9). Pulmonary complications—such as pleural effusion, extensive pulmonary consolidation, atelectasis, and necrotizing pneumonia—were frequently observed in severe MPP (9, 31, 32). Sequela, such as obliterative bronchiolitis and pulmonary fibrosis, may occur after MPP (33, 34).

During clinical practice, we frequently observed deterioration in patients with AdV-MP coinfection, thus analyzed the relationship between AdV and each pathogen, and demonstrated that MP infection predisposed patients to AdV infection (Table 3). In this study, 58% of AdV infected patients were co-infected with MP. MP was reported to be the most prevalent among the pathogens co-infected with AdV (4, 35, 36). In previously studies of Chinese children with lower respiratory tract infection, the AdV-MP coinfection rate was 10%—16% of all AdV infected children (35, 36). The coinfection rate surged to 65% in a recent study by Li et al. using high-throughput sequencing technology, in which 90 cases of severe CAP children experienced fiberscope intervention, and 80% of cases presented pulmonary consolidation (4). The similarly high prevalence of AdV-MP coinfection in this study might be explained by a shift of dominant pathogens over time, along with a high sensitivity of the molecular assay.

Among age, sex, and all studied pathogens, only MP coinfection was proved to be an independent risk factor for AdV-induced severe pneumonia in our study (Table 4). Likewise, Wei et al. compared pneumonia children with MP-AdV coinfection (*n* = 125) to those with single AdV infection (*n* = 171), and found that MP-AdV coinfection was associated with longer fever duration, longer hospital stay, and more pulmonary image findings such as pulmonary consolidation (37). Whereas contrary evidence about image findings was reported by Li et al, who found similar positive rates of MP- and AdV-infection between patients with or without pulmonary consolidation (4). Aside from coinfection, in AdV-induced CAP children, the risk factors for severe pneumonia were reported to be AdV serotype 7 (38, 39), viral load (12), comorbidities (25, 38–40), <2 years old (40), etc. This recognized risk factor of MP coinfection may further aid in predicting prognosis in AdV-infected patients.

Meanwhile, the risk factors for MP-induced severe pneumonia included sex, age, and coinfection with bacteria, CP or AdV (Table 5). Chiu et al. collected clinical data from 59 children with MP pneumonia (MPP), and found that Streptococcus pneumoniae coinfection was more likely in children under 5 years of age and was associated with longer duration of fever and hospital stay (41). Zhou et al. investigated 107 cases of MPP children, and observed that patients with AdV coinfection had longer duration of fever, more extrapulmonary complications and more consolidation (42). Likewise, Gao et al.reported higher illness severity in MPP patients with AdV coinfection (43). The underlying mechanism is far from understood, while a potential synergistic relationship between MP and other pathogens was proposed (44). A recent study detected lung microbiota in BALF, and found that compared to MP monoinfection, MP-AdV coinfection increased intragroup difference albeit a similar species richness, and may shed light on the potential mechanism (45).

Regarding the age and sex, we found that age had a strong influence on the etiology of severe CAP, whereas sex had less influence (Table 2). Notably, we found that patients older than 1 year had a 10-fold higher risk of being infected with MP. The effects of age on etiology confirmed the previous observation, though a direct comparison among clinical studies is not applicable due to different age grouping (15). Contrary to patient age, sex did not affect the incidence of most pathogens except for MP. Nevertheless, the susceptibility of female to MP infection was observed as well in previous studies (5, 46). Considering the effect of pathogens on the incidence of severe CAP, as demonstrated in this study, it implies that sex was not a major factor leading to severe CAP.

This study mainly has two limitations. First, the detection method was not uniform owing to the recently available molecular assay, thus the overall positive rate of infected pathogens are presumably to be underestimated. Moreover, previous antibiotic use was not excluded, and may bias the positive rate of bacteria.

In summary, we detected AdV and MP to be the dominant pathogens in children with severe CAP. We proved that AdV infection and MP infection predisposed each other to develop severe illness. These findings support a potential synergistic relationship between AdV and MP, which may induce disease deterioration and lead to severe pneumonia. However, single center studies and results may not be applicable to other regions, making it difficult to use this information in clinical practice, especially for clinicians in other countries.Further large-scale multicenter clinical studies are still needed, and the immune signaling pathways that may be involved in pathological and physiological processes require extensive basic research to clarify.

## Data Availability

The original contributions presented in the study are included in the article/Supplementary Material, further inquiries can be directed to the corresponding author.
